# Supplementary material for: Association between the GLP1R A316T Mutation and Adolescent Idiopathic Scoliosis in French Canadian and Italian Cohorts
Source: Genes (Basel). 2024 Apr 11;15(4):481. doi: 10.3390/genes15040481 (PMC11050147; doi:10.3390/genes15040481)
Supplement: Supplementary file 1 [file genes-15-00481-s001.zip › genes-2928183-supplementary.pdf]

**Supplementary Table S1.** Association of the *GLP1R* rs10305492 polymorphism with AIS susceptibility in the French-Canadian and Italian cohorts

| | French-Canadian cohort (n=636) | | | | | | | Italian cohort (n=389) | | | | | | |
|---|---|---|---|---|---|---|---|---|---|---|---|---|---|---|
| ***GLP1R*** | **Controls** | | **AIS** | | | | **OR** | **Controls** | | **AIS** | | | | **OR** |
| **rs10305492** | **n** | **(%)** | **n** | **(%)** | **$\chi^2$** | **P value[a]** | **(95% CI)** | **n** | **(%)** | **n** | **(%)** | **$\chi^2$** | **P value[a]** | **(95% CI)** |
| **Genotype** | | | | | 1.70 | 0.193 | | | | | | 0.06 | 0.807 | |
| G/G | 93 | (97.9) | 513 | (94.8) | | | Reference | 216 | (99.1) | 169 | (98.8) | | | Reference |
| A/G | 2 | (2.1) | 28 | (5.2) | | | 2.54 (0.70 – 10.97) | 2 | (0.9) | 2 | (1.2) | | | 1.28 (0.18 – 8.96) |
| **Allele** | | | | | 1.65 | 0.198 | | | | | | 0.06 | 0.804 | |
| G | 188 | (98.9) | 1054 | (97.4) | | | Reference | 436 | (99.5) | 340 | (99.4) | | | Reference |
| A | 2 | (1.1) | 28 | (2.6) | | | 2.50 (0.64 – 10.73) | 2 | (0.5) | 2 | (0.6) | | | 1.28 (0.20 – 8.22) |

Allelic and genotypic distributions of the polymorphism was assessed in AIS patients and healthy controls separately in the French-Canadian and Italian cohorts. AIS: Adolescent idiopathic scoliosis, OR: Odds ratio, CI: confidence interval. [a] Pearson's chi-square test ($\chi^2$).
